# Chronicity and its impact on workers’ health: a call for concrete actions

**DOI:** 10.1590/0034-7167.202376suppl301

**Published:** 2023-12-04

**Authors:** João Vitor Andra, Cristiane Aparecida Silveira, Fábio de Souza Terra

**Affiliations:** I Universidade Federal de Alfenas. Alfenas, Minas Gerais, Brazil

The relationship between chronicity and work has been a growing concern in contemporary society. As the number of chronic illnesses increases around the world, it is critical to understand how these conditions impact workers’ health^(1)^. In this editorial, we will explore the impacts of chronicity on workers’ health, highlighting data from the Global Burden of Disease (GBD) and specific evidence from the Brazilian context, especially in relation to absences from work.

Chronic diseases, such as diabetes, cardiovascular diseases, chronic respiratory diseases, and mental disorders, account for a significant portion of GBD. These conditions can have a significant impact on people’s ability to carry out their activities of daily living, including work. It is estimated that chronic diseases account for more than 70% of deaths worldwide. Furthermore, it is noted that, in 2019, these diseases were responsible for 83.3% of the years lived with disability, which, consequently, contributes to absence from work^(2)^.

In the work context, chronicity can affect both workers and organizations. People with chronic illnesses often face difficulties in dealing with the symptoms and associated physical and mental limitations, which can lead to an increase in presenteeism, absenteeism and sick leave. Furthermore, worker productivity can be reduced due to chronic fatigue, compromising not only individual well-being but also the overall performance of institutions^(3)^.

In Brazil, the numbers related to chronicity and absence from work are equally worrying. According to data from the Brazilian National Social Security Institute (INSS - *Instituto Nacional do Seguro Social*), chronic illnesses represent one of the main causes of sick leave in the country. In the last survey, carried out in 2021, it was estimated that around 300 thousand absences were recorded due to chronic conditions, generating significant socioeconomic impacts^(4)^.

It is essential to highlight that the relationship between chronicity and work not only affects workers’ health, but also the sustainability of health systems and the economy in general. The direct and indirect cost of this interaction between illness and work is substantial, involving medical expenses, reduced productivity and early retirement. Therefore, it is imperative that comprehensive measures be adopted to address this issue^(5)^, mainly the creation of spaces and educational activities in occupational health, thus seeking an environment for team engagement and sharing of experiences and knowledge^(6)^.

Given this worrying scenario, a joint effort is needed to deal with chronicity and its impact on workers’ health. The following measures can be considered:

1. Health promotion and illness prevention: invest in health promotion and chronic disease prevention programs in the workplace, focusing on education, healthy eating, regular physical activity and stress management.
2. Work environment adaptation and/or healthy environment construction: promote healthy work environments adapted to the needs of workers with chronic illnesses, ensuring accessibility and reasonable adjustments to facilitate their full and productive participation.
3. Mental health care: prioritize workers’ mental health by providing adequate psychological support, stress management programs and awareness of the importance of work-life balance.
4. Policies and legislation: establish policies and regulations that encourage the inclusion and avoid discrimination of workers with chronic illnesses, in addition to offering support for reintegration into work after a leave of absence.

Finally, it is essential that concrete actions are implemented, such as preventive and support measures, to ensure that workers with chronic illnesses have equitable opportunities to fully participate in the labor market, resulting in a satisfactory quality of working life. By adopting comprehensive, evidence-based approaches, we can promote their health and well-being, strengthening the sustainability and productivity of organizations.
